# Case Report: Persistent Moderate-to-Severe Creatine Kinase Enzyme Activity Elevation in a Subclinical Dog

**DOI:** 10.3389/fvets.2021.757294

**Published:** 2021-10-25

**Authors:** Melissa Gunther, Jared A. Jaffey, Jason Evans, Christopher Paige

**Affiliations:** ^1^Department of Specialty Medicine, College of Veterinary Medicine, Midwestern University, Glendale, AZ, United States; ^2^Valley Veterinary Cardiology, Scottsdale, AZ, United States

**Keywords:** CK, muscular dystrophy, hyperCKemia, myopathy, canine

## Abstract

A 4-year-old, male-castrated, mixed breed dog was presented for a routine wellness examination at which time a moderate increase in serum creatine kinase (CK) enzyme activity (hyperCKemia) (15,137 IU/L; reference interval 10–200 IU/L), and moderate increases in alanine transaminase and aspartate aminotransferase enzyme activities were first identified. There was no history of clinical abnormalities (e.g., lethargy, lameness, anorexia, dysphagia, weakness, gait abnormalities, or exercise intolerance) and the physical examination was unremarkable. The dog was screened for several relevant potential infectious diseases known to cause inflammatory myopathies and was treated empirically with clindamycin. The serum total CK enzyme activity remained increased, which prompted recommendations for an echocardiogram, electromyogram (EMG), and muscle biopsy acquisition. The echocardiogram and electrocardiographic monitoring were unremarkable. The EMG and muscle biopsies were declined by the owner. The dog was evaluated several times in the subsequent 5 years and remained subclinical with unremarkable physical examinations despite a persistent moderate-to-severe hyperCKemia. Differential diagnoses considered most likely in this dog were an occult/latent hereditary muscular dystrophic disorder or idiopathic hyperCKemia, a phenomenon not yet reported in the veterinary literature. This report describes for the first time, clinical and diagnostic features of a subclinical dog with persistent moderate-to-severe hyperCKemia.

## Background

Creatine kinase (CK) catalyzes the reversible transfer of phosphate residues for the storage and utilization of energy in skeletal muscle and has a minor role in the redistribution of energy from the mitochondria to the cytoplasm in various tissues (1). This enzyme can be found in all tissues but tends to have the greatest activity in organs with high energy requirements including skeletal muscle, cardiac muscle, and brain tissue (1). Creatine kinase is predominately cytosolic and leakage from trauma or disease causes increased CK activity in blood (hyperCKemia), making it a specific biomarker for muscle fiber damage (1, 2). Total CK activity in blood represents the cumulative measurement of skeletal muscle (CK-MM), cardiac muscle (CK-MB), and brain tissue (CK-BB) isoenzyme activity (1, 3). While the total CK activity in blood is a collective product of various isoenzyme fractions, CK-MM is the predominant isoenzyme in dogs, cats, and humans (3–5).

The magnitude and chronicity of hyperCKemia is an indication of severity and activity of disease. The half-life of serum CK in dogs is short at 2.5 h; therefore, it should decrease rapidly after cessation of the inciting injury (6). Transient mild to moderate hyperCKemia can be identified following external muscle damage from various causes such as prolonged recumbency, intramuscular injections, or trauma (2). The degree of hyperCKemia can also provide insight into the source and type of disease. HyperCKemia in dogs with focal inflammatory myopathies, congenital myopathies, or primary neurologic disease is either absent or mild (0–2,000 IU/L) (2). Moderate hyperCKemia (2,000–20,000 IU/L) can be identified in dogs with generalized inflammatory myopathies, while severe CK derangements (> 20,000 IU/L) are reserved for dogs with necrotizing myopathies (e.g., drugs, toxins, excessive exertion, hyperthermia, infectious diseases, etc.), or muscular dystrophic diseases (2). Elevation of CK has also been reported with severe hypothyroidism (7).

Generalized or focal clinical signs are typically demonstrated in dogs with diseases that result in moderate-to-severe hyperCKemia with the exception of hereditary muscular dystrophic disorders (2, 8). Dogs with muscular dystrophies can be subclinical in the occult phase of disease but develop progressive muscle weakness and wasting over time (2). Currently, management and prognosis of dogs with persistently increased serum CK enzyme activity and minimal or absent clinical signs is unclear. To the authors' knowledge, there have been no published reports describing this in dogs. Therefore, the objectives of this report were to provide a thorough clinical description of a dog with persistent moderate-to-severe hyperCKemia that remained subclinical for 5 years.

## Case Presentation

**Figure 1:**
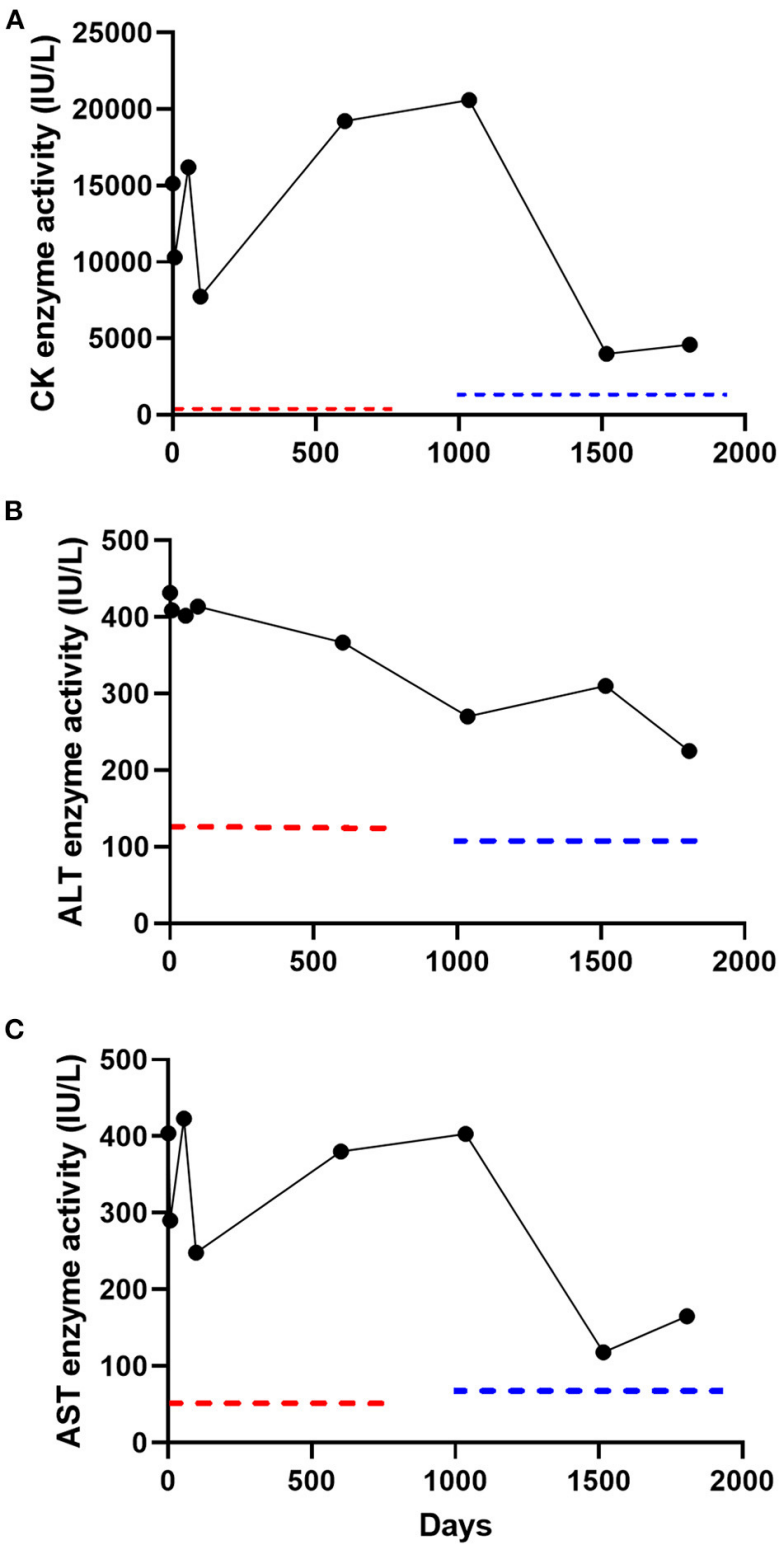
Serial serum creatine kinase (CK) **(A)**, alanine transaminase (ALT) **(B)**, and aspartate aminotransferase (AST) **(C)** enzyme activity measurements in a subclinical dog over 1,807 days (5 years). Two reference laboratories were used over the 5-year follow-up period (laboratory A; day 1–602; laboratory B, day 1,036–1,807). Reference intervals for laboratory A included CK (10–200 IU/L), ALT (18–121 IU/L), and AST (16–55 IU/L) and laboratory B included CK (59–895 IU/L), ALT (12–118 IU/L), and AST (15–66 IU/L). The dotted red and blue lines denote the upper limit of the reference interval for laboratory A and laboratory B, respectively.

A 4-year old, male-castrated, mixed breed dog was presented to a primary care veterinarian for a routine wellness examination (day 1). The dog was adopted 7-months prior and the previous medical history was unknown. The owner reported that the dog exercised daily without limitation, lived indoors only, and had no history of trauma. The dog was fed a commercial kibble diet and not administered any medications or supplements. The physical examination, which included thorough neurologic, musculoskeletal, and cardiopulmonary evaluations, was unremarkable. A complete blood count, serum biochemistry, symmetric dimethylarginine (SDMA), and urinalysis were performed. Clinically relevant abnormalities on the serum biochemistry performed at that time included a severe hyperCKemia (15,137 IU/L; reference interval 10–200 IU/L), and moderate increases in alanine transaminase (ALT; [432 IU/L; reference interval 180–121 IU/L]) and aspartate aminotransferase (AST; [404 IU/L; reference interval 16–55 IU/L]) enzyme activities. The complete blood count, SDMA, and urinalysis were unremarkable. A serum biochemical profile was repeated 7-days later, which revealed persistently increased serum CK activity (10,301 IU/L) as well as ALT (409 IU/L) and AST (290 IU/L) enzyme activities (Figure 1). Recommendations for additional diagnostic testing were declined by the owner in favor of empirical treatment for potential toxoplasmosis/neosporosis with clindamycin (22.7 mg/kg, PO, q12 h for 14 days).

The dog was presented for evaluation on day 55. The owner reported no clinical changes in the dog and the physical examination remained unremarkable. A serum biochemistry was repeated and revealed relatively unchanged serum CK, ALT, and AST enzyme activities (Figure 1). A commercial ELISA-based kit (4DX SNAP Plus Test kit, IDEXX Laboratories Inc., Westbrook, ME) was negative for detection of antibodies for *Ehrlichia* sp., *Anaplasma phagocytophilum, Anaplasma platys, Borrelia burgdorferi* C6 peptide, and *Dirofilaria immitis* antigen. Differential diagnoses considered by the primary care veterinarian at that time included infectious polymyositis (*Toxoplasma gondii* or *Neospora canis* [potentially resistant to clindamycin], *Hepatozoon canis* or *americanum*), immune-mediated polymyositis, paraneoplastic polymyositis, congenital myopathy, or a hereditary muscular dystrophic disorder. The following diagnostic tests were recommended; skeletal muscle biopsy, electromyogram (EMG), echocardiogram, serum antinuclear antibodies, and additional infectious disease testing. The owner declined these recommendations because the dog was subclinical and instead opted for an additional course of clindamycin (22.7 mg/kg, PO, q12 h for 30 days). The dog was presented for evaluation on day 97. Again, the owner reported no abnormalities and the dog had an unremarkable physical examination. A serum biochemistry revealed relatively unchanged serum CK, ALT, and AST enzyme activities (Figure 1). All additional diagnostic testing recommendations were declined and the dog was discharged without medications.

The dog was presented to the primary care veterinarian for a wellness examination on day 602. The owner reported no abnormalities since the dog was last evaluated (day 97). The dog continued to exercise daily without limitation. Physical examination was unremarkable. There was no pain elicited with muscle palpation. A complete blood count, serum biochemistry, and urinalysis were performed. The serum biochemistry revealed persistence in severe hyperCKemia and mild to moderate increases in ALT and AST enzyme activities (Figure 1). The complete blood count and urinalysis were unremarkable. The owner declined additional diagnostic tests or empirical therapies at that time because the dog did not demonstrate any clinical abnormalities.

The dog was then presented to the Midwestern University Companion Animal Clinic (MWU-CAC) on day 1,036 for a second opinion regarding the severe hyperCKemia and mild-to-moderate increase in ALT and AST enzyme activities. The dog remained subclinical and exercised daily without limitation. A physical examination performed by a boarded small animal internist (JAJ) was unremarkable. The dog had a normal muscle conditioning score and pain was not elicited with deep palpation of musculature. A neurologic examination performed by a boarded neurologist (JE) revealed the dog was bright, alert and responsive. The dog was ambulatory with no signs of stilted gait, lameness or paresis. There was no overt exercise intolerance after several minutes of walking and trotting. Postural reactions, spinal reflexes and cranial nerves were normal. Serum biochemistry revealed an unchanged severe hyperCKemia and persistent mild-to-moderate derangements in ALT and AST enzyme activities (Figure 1). The dog was negative for detection of antibodies (IgM and IgG) for *Toxoplasma gondii* or *Neospora canis* (Protatek Reference Laboratory) and nucleic acids associated with *Hepatozoon* spp. (Texas A&M Veterinary Medical Diagnostic Laboratory) were not detected. An abdominal ultrasonogram performed by a boarded small animal internist (JAJ) trained in ultrasonography was unremarkable. Differential diagnoses considered at that time in order of likelihood was hereditary muscular dystrophy, and less likely necrotizing myopathy, generalized or focal inflammatory myopathy. Additional diagnostic tests including EMG, echocardiogram, or skeletal muscle biopsies were offered but declined by the owner.

The dog was evaluated on day 1,516 at the MWU-CAC for a pre-anesthetic examination for a dental prophylactic procedure. The owner reported no abnormalities since the dog was last evaluated (day 1,036) and the physical examination remained unremarkable. The dog had no muscle loss and remained active at home. A serum biochemistry revealed an improved, but persistent moderate hyperCKemia and mild-to-moderate increase in ALT and AST enzyme activities (Figure 1). A complete blood count and urinalysis were unremarkable. Next, an echocardiogram and electrocardiogram were performed by a boarded cardiologist (CP) in light of the persistent and unknown cause of severe hyperCKemia to ensure the dog did not have clinically relevant heart disease before undergoing anesthesia. Based on the echocardiographic findings, ACVIM Stage B1 degenerative valvular disease was detected, and demonstrated mild mitral and mild tricuspid regurgitation (9). The global systolic function appeared preserved. During the echocardiogram, Lead II electrocardiographic monitoring revealed a normal sinus rhythm with no evidence of ectopy.

The last in-hospital recheck examination took place on day 1,807 (5 years from initial evaluation). The owner reported no clinical abnormalities and the physical examination was unremarkable (Supplementary Video). The neurological examination was again normal. A serum biochemistry revealed a moderate hyperCKemia (4,601 IU/L) and mild-to-moderate increases in ALT (225 IU/L) and AST (165 IU/L) enzyme activity (Figure 1). Electrophoretic identification of serum CK isoenzymes (Antech Diagnostics, Irvine, CA, USA) revealed that CK-MM was the predominant electrophoretic fraction (83.7%), followed by CK-MB (10.3%) and CK-BB (6.0%). No injections were administered to the dog at or near the time serum total CK was measured at any of the examination time-points.

## Discussion

Persistent increases in serum CK enzyme activity in dogs is commonly linked to disease categories such as necrotizing myopathies, generalized or focal inflammatory myopathies, and congenital or hereditary muscle disorders (2, 8). The magnitude and chronicity of increased serum CK enzyme activity is usually an indication of severity and activity of muscle pathology (2). This concept can be used to facilitate prioritization of potential differential diagnoses. Severe increases in serum CK enzyme activity (i.e., > 20,000 IU/L) are usually associated with necrotizing myopathies or hereditary muscular dystrophic disorders (2, 10, 11). Necrotizing myopathies are generally associated with ≥ 1 clinical sign such as myalgia, fever, stilted gait, generalized weakness, decreased spinal reflexes, exercise intolerance, anorexia, lethargy, pigmenturia, or dysphagia and may be precipitated by factors such as drugs, toxins, snake bites, insect stings, ischemic myopathy, electrolyte disorders (e.g., hypokalemia), endocrinopathies, excessive exertion, hyperthermia, infectious diseases or are idiopathic (2, 10–12). Hereditary muscular dystrophies could be associated with minimal clinical signs early in life but generally progress (13, 14). Generalized and focal inflammatory myopathies are usually associated with moderate (i.e., 2,000–20,000 IU/L) and mild (i.e., 0–2,000 IU/L) increases in serum CK enzyme activity, respectively (2, 8). Generalized inflammatory myopathies demonstrate similar clinical signs as necrotizing myopathies and can have an infectious (e.g., *Neospora caninum, Hepatozoon* spp., *Toxoplasma gondii, Ehrlichia canis, Dirofilaria imminits*) etiology or can be immune-mediated or paraneoplastic (8, 15–19). Other infectious causes not considered in this case because of a lack of endemicity or exposure include *Leishmania infantum, Trichinella spiralis*, and sarcocystosis (19, 20). Focal inflammatory myopathies including masticatory muscle myositis and extraocular myositis manifest with clinical signs reflective of the involved muscles, which were not observed in our case (11).

The utilization of blood AST and ALT enzyme activity is a valuable adjunct to the clinical diagnosis of neuromuscular diseases in both dogs and humans (8, 21, 22). These enzymes are normally located in myofibers and are released with muscle damage (1, 2). Interestingly, there tends to be a high correlation of CK, AST, and ALT ratios at all stages of the disease process (22). While blood AST and ALT enzyme activity are clearly valuable as a diagnostic aid in suspected neuromuscular disorders, the enzymes lack organ specificity as they are also found in other organs such as cardiac muscle, liver, and erythrocytes (2). Aspartate aminotransferase enzyme activity generally tends to be elevated to a higher magnitude than ALT activity in neuromuscular disorders; however, this pattern is not definite (21, 23). These values can be within their respective reference intervals with myopathic disorders and blood activity of ALT can be higher than AST (21, 23). Serum AST and ALT enzyme activities proved valuable as adjunctive support for our clinical suspicion of neuromuscular disease in the dog reported here. These results also highlight that serum activity of ALT can be higher than AST in these diseases and can oscillate over time.

Serum CK activity was 2–3 times higher at baseline in the dog reported here compared to results obtained 5 years later. It has been reported that blood CK activity in humans with idiopathic hyperCKemia can oscillate over time (22). However, the definition of hyperCKemia is typically a persistent blood CK result that is 1.5 times above the upper limit of the reference interval (24). The dog in our report continued to meet this criterion throughout the 5-year follow-up.

Possible differential diagnoses of the case reported here included necrotizing myopathy, generalized inflammatory myopathy, or hereditary muscular dystrophy; however, only the latter disorder can be found in subclinical dogs (10, 11, 13). While dogs with hereditary muscular dystrophies can initially be subclinical, most will develop progressive clinical signs such as muscle weakness, atrophy, or hypertrophy as well as exercise intolerance, gait abnormalities, hypertonicity, stiffness, or dysphagia. The adult age that hyperCKemia was first identified and the lack of clinical signs despite persistent severe hyperCKemia for 5 years in the dog reported here makes muscular dystrophic disorders such as dystrophinopathies, laminin α2 deficiency, sarcoglycanopathies, caveolin-3 trafficking alterations, calpain-3 deficiency, and collagen VI muscular dystrophy, unlikely (14, 25–28). Similarly, the lack of development of any clinical signs also made an inflammatory process unlikely. However, compared to human medicine, there is a paucity of knowledge about the extent of hereditary muscular dystrophies in veterinary medicine. Other forms of hereditary muscular dystrophies could exist in dogs, as they do in humans, where progression is slow and clinical signs are subtle.

Muscle biopsies evaluated using a standard panel of histochemical stains and reactions (including fiber typing) as well as advanced immunohistochemical analysis for expression of relevant muscle proteins like dystrophin, dystrophin-associated proteins, laminins, and other proteins would have provided the best opportunity to identify the causal disease in the dog reported here but was declined by the owner (14). In humans, management of patients with increased serum CK enzyme activity and minimal or absent symptoms is a clinical dilemma. Most adult muscular dystrophies and metabolic myopathies have no treatment and their clinical course is often benign, whereas juvenile onset of these disorders tend to be more severe and progressive (24). In addition, a review of three large human studies revealed that only 28% of patients with asymptomatic hyperCKemia obtained a specific diagnosis after combined muscle biopsy, EMG, and nerve conduction studies (29–31). Therefore, physicians often weigh the value of expensive and invasive diagnostic tests to determine a specific disease against the limited yield and treatment options. The case reported here suggests that, like humans, rigorous diagnostic investigations may not be necessary in subclinical adult dogs with chronic hyperCKemia.

Idiopathic hyperCKemia is a rare condition in humans characterized by persistent increase of serum CK enzyme activity and no clinical, neurophysiological, or histopathological evidence of neuromuscular disease (24, 32). Humans with this condition have a favorable long-term prognosis. Only 3% (2/78) of patients with an original diagnosis of idiopathic hyperCKemia from two studies went on to develop a neuromuscular disorder in the follow-up period with one each of chronic idiopathic axonal polyneuropathy and limb-girdle dystrophy (33, 34). Idiopathic hyperCKemia is another reasonable differential diagnosis to explain the chronic subclinical hyperCKemia in the dog reported here; however, without electrophysiologic testing and muscle biopsies, other causes cannot be ruled out. This phenomenon has not been reported or even discussed as a possible differential diagnosis in the veterinary literature.

## Conclusion

In conclusion, this report provides the first description of the clinical and diagnostic features of a subclinical dog with persistent moderate-to-severe hyperCKemia for 5 years. These results suggest that in the absence of clinical signs, persistent hyperCKemia can be a benign clinicopathologic abnormality in some dogs.

## Data Availability Statement

The original contributions presented in the study are included in the article/Supplementary Material, further inquiries can be directed to the corresponding author.

## Ethics Statement

Ethical review and approval was not required for the animal study because this case report used historical information from the medical record and therefore did not require owner consent or IACUC approval.

## Author Contributions

MG: writing and editing manuscript and review of final submission. JJ: management of case, collection of data, writing and editing of manuscript, figure preparation, and review of final submission. JE and CP: management of case, writing and editing of manuscript, and review of final submission. All authors contributed to the article and approved the submitted version.

## Conflict of Interest

The authors declare that the research was conducted in the absence of any commercial or financial relationships that could be construed as a potential conflict of interest.

## Publisher's Note

All claims expressed in this article are solely those of the authors and do not necessarily represent those of their affiliated organizations, or those of the publisher, the editors and the reviewers. Any product that may be evaluated in this article, or claim that may be made by its manufacturer, is not guaranteed or endorsed by the publisher.
